# Sense of Purpose in Life and Subsequent Physical, Behavioral, and Psychosocial Health: An Outcome-Wide Approach

**DOI:** 10.1177/08901171211038545

**Published:** 2021-08-18

**Authors:** Eric S. Kim, Ying Chen, Julia S. Nakamura, Carol D. Ryff, Tyler J. VanderWeele

**Affiliations:** 1 Department of Psychology, University of British Columbia, Vancouver, British Columbia, Canada; 2 Human Flourishing Program, Institute for Quantitative Social Science, Harvard University, Cambridge, MA, USA; 3 Lee Kum Sheung Center for Health and Happiness, Harvard T.H. Chan School of Public Health, Boston, MA, USA; 4 Department of Epidemiology, Harvard T.H. Chan School of Public Health, Boston, MA, USA; 5 Department of Psychology, University of Wisconsin, Madison, WI, USA; 6 Institute on Aging, University of Wisconsin, Madison, WI, USA; 7 Department of Biostatistics, Harvard T.H. Chan School of Public Health, Boston, MA, USA

**Keywords:** epidemiology, purpose in life, sense of purpose, psychological well-being, well-being

## Abstract

**Purpose:**

Growing evidence indicates that a higher sense of *purpose* in life (*purpose*) is associated with reduced risk of chronic diseases and mortality. However, epidemiological studies have not evaluated if change in *purpose* is associated with subsequent health and well-being outcomes.

**Design:**

We evaluated if positive change in *purpose* (between t_0_; 2006/2008 and t_1_;2010/2012) was associated with better outcomes on 35 indicators of physical health, health behaviors, and psychosocial well-being (at t_2_;2014/2016).

**Sample:**

We used data from 12,998 participants in the Health and Retirement study—a prospective and nationally representative cohort of U.S. adults aged >50.

**Analysis:**

We conducted multiple linear-, logistic-, and generalized linear regressions.

**Results:**

Over the 4-year follow-up period, people with the highest (versus lowest) purpose had better subsequent physical health outcomes (e.g., 46% reduced risk of mortality (95% CI [0.44, 0.66])), health behaviors (e.g., 13% reduced risk of sleep problems (95% CI [0.77, 0.99])), and psychosocial outcomes (e.g., higher optimism (β = 0.41, 95% CI [0.35, 0.47]), 43% reduced risk of depression (95% CI [0.46, 0.69]), lower loneliness (β = −0.35, 95% CI [−0.41, −0.29])). Importantly, however, purpose was not associated with other physical health outcomes, health behaviors, and social factors.

**Conclusion:**

With further research, these results suggest that sense of purpose might be a valuable target for innovative policy and intervention work aimed at improving health and well-being.

## Purpose

Meeting the unique needs of our rapidly growing older adult population throughout the world is considered a next global public health opportunity.^1^ For this effort, identifying factors that foster health and well-being is crucial. While much effort has focused on identifying risk factors of disease, investigators are increasingly seeking potentially modifiable health assets that uniquely enhance a person’s ability to foster healthy behaviors and physical health.^2-7^ A sense of purpose in life is one promising candidate; it is viewed as a central component of well-being and refers to the extent that people see their lives as having meaning, a sense of direction, and goals.^4,8-12^ It is shaped by social structural factors and changing life circumstances, with ongoing work exploring what the determinants of purpose are,^13-15^ and whether it can be intervened upon, issues to which we return after reporting our findings.^4,16-19^ A growing body of research has also observed that having a higher sense of purpose is associated with better: health behaviors (e.g., increased physical activity, increased preventive healthcare use, healthier sleep, reduced drug misuse),^14,20-26^ biological functioning (e.g., reduced allostatic load, reduced inflammation),^27-29^ better physical functioning, and reduced risk of disease (e.g., lower risk of cardiovascular disease and cognitive impairment),^14,29-35^ and mortality.^31^

These pioneering studies have contributed substantially to the literature. Although many newer studies in this area address limitations brought to light by advances in the field of causal inference, many prior studies remain limited in several ways. First, some studies used data from small and specific subpopulations (e.g., college students and patient groups), which may not generalize to older adults or healthy populations. Second, some studies did not adequately account for potential confounders (e.g., baseline health, psychological distress). Third, most longitudinal studies did not control for pre-baseline outcomes, thereby failing to address reverse causality concerns. Fourth, some studies used limited assessments of purpose in life (e.g., single-item measures). Fifth, few, if any, longitudinal studies have controlled for purpose in life in the pre-baseline wave, which allows researchers to ask a slightly different question—how changes in purpose (“incident exposure”) affect health.

In this era of translational research, interventionists and policy makers are seeking answers to a different question that past studies have not addressed. What health and well-being outcomes might we observe if purpose was intervened upon? To begin addressing this question, we used an outcome-wide analytic approach,^36^ and performed analyses to examine whether positive change in purpose at baseline was associated with better subsequent health and well-being across 35 separate outcomes (indicators of: physical health, health behaviors, psychological well-being, psychological distress, and social well-being) in a large, prospective, and nationally representative sample of adults aged over 50. These outcomes were chosen because they are frequently included in the conceptualization of key gerontological models that characterize the antecedents, processes, and outcomes that foster people’s ability to age well.^37-41^ In these analyses we controlled for: the exposure (purpose) in the pre-baseline wave, a robust range of potential confounders, and all outcomes. This helps condition on or remove the potential accumulating effects that past purpose had on health/well-being in the past, thus allowing us to evaluate the effects of *change in* purpose, and provides better estimates of the outcomes we might expect observe if purpose was intervened upon. To the best of our knowledge, no existing studies in this area have used this approach.

## Methods

### Sample

We used data from the Health and Retirement Study (HRS), an ongoing nationally representative panel study of U.S. adults aged >50, and it surveys participants every 2 years. Starting in 2006, study staff visited a randomly-selected 50% of HRS study participants for an enhanced face-to-face (EFTF) interview. The remaining 50% of participants were assessed in 2008. After the interview, respondents were given a self-administered psychosocial questionnaire,^42^ which they completed and returned by mail to the University of Michigan. The response rate for this psychosocial questionnaire was 88% in 2006 and 84% in 2008.^42^ To increase our sample size and statistical power, we combined data from both subcohorts. The sample was restricted to individuals who completed the psychosocial questionnaire at baseline because more than half of study outcomes were included in this assessment; this resulted in a final sample size of 12,998.

Our study used data from 3 time points (t_0_, t_1_, and t_2_). All covariates were assessed in the pre-baseline wave (t_0_, 2006/2008). This choice was made because controlling for potential confounders in the pre-baseline wave helps alleviate the concern as to whether the variable is a confounder or a mediator. The exposure, purpose in life, was then assessed 4 years later in the baseline wave (t_1_, 2010/2012), while all outcomes were subsequently assessed another 4 years later in the outcome wave (t_2_, 2014/2016). The HRS website (http://hrsonline.isr.umich.edu) provides extensive documentation about the study. Because the present study used de-identified, publicly available data, the ethics board at the University of British Columbia exempted it from review.

### Measures

#### Sense of purpose in life

Purpose was assessed at baseline (t_1_; 2010/2012) using the 7-item purpose in life subscale of the Ryff Psychological Well-Being Scales,^43^ previously validated in a nationally representative sample of adults. On a 6-point Likert scale, respondents rated the degree to which they endorsed items such as, “I have a sense of direction and purpose in my life.” The mean of all items was taken to create a scale with scores ranging from 1 to 6 where higher scores reflected higher sense of purpose (Cronbach α = 0.76). Following HRS protocol, if respondents completed 4 or more (of 7 items), a purpose score was derived. To examine threshold effects, we created quartiles based on the baseline distribution of purpose scores in the sample.

#### Outcomes

Thirty-five outcomes were assessed in the outcome wave (t_2_; 2014/2016), including: physical health factors (all-cause mortality, number of chronic conditions: diabetes, hypertension, stroke, cancer, heart disease, lung disease, arthritis, overweight/obesity, physical functioning limitations, cognitive impairment, chronic pain, self-rated health), health behaviors (heavy drinking, smoking, physical activity, sleep problems), psychological well-being (positive affect, life satisfaction, optimism, purpose in life, mastery, health mastery, financial mastery), psychological distress (depression, depressive symptoms, hopelessness, negative affect, perceived constraints) and social factors (loneliness, living with a spouse/partner, and contact with: children, other family, and friends). The appendix provides information about how each of these variables were assessed and the following HRS guides provide extensive documentation about each of these measures (see Supplementary Text 1 for further details).^42,44,45^

#### Covariates

All covariates were assessed by self-report in the pre-baseline wave (t_0_; 2006/2008, the closest wave pre-baseline to the exposure assessment. Potential confounders included: sociodemographic factors (age, sex, race/ethnicity (Caucasian, African-American, Hispanic, Other), marital status (married/not married), income (<$50,000; $50,000-$74,999; $75,000-$99,999; ≥$100,000), total wealth (based on quintiles of the score distribution in this sample), educational attainment (no degree, GED or high school diploma, college degree or higher), employment status (yes/no), health insurance (yes/no), geographic region (Northeast, Midwest, South, West), religious service attendance (none, <1x a week, ≥1x per week), personality factors (openness, conscientiousness, extroversion, agreeableness, neuroticism), and childhood abuse (yes/no). To reduce the possibility of reverse causation, we controlled for pre-baseline values of all outcome variables in our models.^36^ The appendix and HRS guides provide information about each of these assessments. To evaluate *changes in* purpose (conditional on the past), we also controlled for purpose in the pre-baseline wave (t_0_; 2006/2008); doing so helps rule out reverse causation and potential unmeasured confounding.^46^

### Analysis

We used an outcome-wide analytic approach,^36^ and it contains several features that are not widely used outside of biostatistics and causal inference. Thus, we summarize those features here. First, if we assess potential confounders in the same timepoint as the exposure (t_1_), it remains unclear if they are confounders or mediators. That is why we adjust for potential confounders in the pre-baseline wave (t_0_). Second, we adjust for a broad array of potential confounders variables because this enhances our ability to strive toward “exchangeability” and “no unmeasured confounding,” which in turn enhances our ability to make causal inference.^47,48^ Third, we adjust for all outcome variables in the pre-baseline wave (t_0_) to reduce potential reverse causality. Fourth, to evaluate potential “change” in purpose we adjust for purpose in the pre-baseline wave (t_0_). Doing so helps “hold constant” pre-baseline levels of purpose. Therefore, those who have the highest levels of purpose in the pre-baseline wave (t_0_) and continue having the highest levels of purpose in the baseline wave (t_1_) contribute to the final estimate. However, the estimates produced from this analysis also corresponds to those who started in the lowest levels of purpose in the pre-baseline wave (t_0_) and then moved to the highest levels of purpose in the baseline wave (t_1_). Thus, readers are able to evaluate how *change* in purpose (between t_0_ and t_1;_ see Supplementary Table 1 for further details), are associated with subsequent health and well-being outcomes (at t_2;_ see Supplementary Text 2 for further details). Adjusting for pre-baseline levels of purpose (t_0_) also has several other advantages including helping reduce risk of reverse causality by “removing” the accumulating effects that purpose already had on outcomes in the past (“prevalent exposure”), and allowing readers to instead focus on the effects of *change* in purpose (“incident exposure”) on outcomes. In our tables, we marked multiple p-value thresholds because different investigators often use different standards in interpreting evidence. For ease of reviewing results, the tables include p-value thresholds of: p < 0.05, p < 0.01, or a Bonferroni correction to account for multiple testing (p = 0.05/35 outcomes = p < 0.001). In our results section, we comment on traditional 0.05 p-value threshold (without Bonferroni correction), but in all cases we also provide 95% confidence intervals which give what are often considered preferable assessments of uncertainty since all thresholds are ultimately arbitrary.

#### Additional analyses

We conducted several additional analyses. First, we performed sensitivity analysis using E-values to assess the robustness of an exposure-outcome association to unmeasured confounding by assessing the minimum strength that an unmeasured confounder must have on the risk ratio scale (with both the exposure and the outcome) to explain away the association.^49^ Second, we reanalyzed all models using a reduced list of potential confounders which are more conventionally used in the social and behavioral sciences (e.g., sociodemographics factors and depressive symptoms) to evaluate how similar (or different) our results were compared to past research in the purpose-health/well-being area. Third, we reanalyzed the main models, but removed people who had history of a given condition at baseline (e.g., for the stroke analyses, we removed people who had stroke in the past). Fourth, we reanalyzed all models using only complete-cases.

#### Multiple imputation

When conducting complete-case analyses we had missing data for the exposure (2.85%), covariates (up to 23.73%), and outcomes (up to 38.40%), which ultimately led to a 35.74% to 57.45% drop in sample size, depending on the outcome being evaluated. Thus, we imputed missing data for the exposure, covariates, and outcomes using an imputation by chained equations procedure by generating 5 datasets, as it provides a more flexible approach than other methods of handling missing data.^50^ All analyses were conducted in Stata (Version 16.1).

## Results

At the pre-baseline wave, when all the potential confounders were assessed, the average age of respondents was 65 years old (*SD* = 10), primarily women (59%), and tended to have a high school education (55%). Participants also reported being White (74%), Black (14%), Hispanic (9%), and “Other” (3%). The distribution of sociodemographic and health characteristics was similar across purpose quartiles, but there were some key differences. For example, those in the highest (versus lowest) purpose quartile were more educated (e.g., 35% versus 19% with ≥college degree) and had a lower prevalence of smoking (e.g., 9% vs. 15% were current smokers) and depression (e.g., 5% versus 25%). Table 1 describes the distribution of covariates by quartile of purpose for the other variables.

**Table 1.** Characteristics of Participants at Baseline by Quartiles of Sense of Purpose in Life (N = 9,977).^a,b,c^

Participant characteristics	Purpose in life															
	Quartile 1 (*N* = 2,706)				Quartile 2 (*N* = 2,443)				Quartile 3 (*N* = 2,723)				Quartile 4 (*N* = 2,105)			
	n	%	M	SD	n	%	M	SD	n	%	M	SD	n	%	M	SD
Sociodemographic Factors																
Age (yr; range: 46-96)			68.0	9.7			67.4	9.2			66.8	8.9			65.8	8.7
Female (%)	1617	59.8			1465	60.0			1611	59.2			1264	60.1		
Race/Ethnicity (%)																
White	2091	77.3			1969	80.6			2236	82.2			1626	77.2		
Black	280	10.4			262	10.7			270	9.9			298	14.2		
Hispanic	262	9.7			163	6.7			170	6.3			135	6.4		
Other	73	2.7			49	2.0			46	1.7			46	2.2		
Married (%)	1642	60.7			1614	66.1			1926	70.7			1518	72.2		
Income (%)																
<$50,000	1847	68.3			1419	58.1			1344	49.4			955	45.4		
$50,000-$74,999	395	14.6			396	16.2			522	19.2			339	16.1		
$75,000-$99,999	200	7.4			227	9.3			312	11.5			256	12.2		
≥$100,000	264	9.8			401	16.4			545	20.0			555	26.4		
Total Wealth (%)																
1st Quintile	737	27.2			485	19.9			423	15.5			281	13.4		
2nd Quintile	629	23.2			502	20.6			494	18.1			367	17.4		
3 rd Quintile	525	19.4			496	20.3			552	20.3			415	19.7		
4th Quintile	441	16.3			479	19.6			622	22.8			494	23.5		
5th Quintile	374	13.8			481	19.7			632	23.2			548	26.0		
Education (%)																
< High School	640	23.7			380	15.6			326	12.0			228	10.9		
High School	1549	57.3			1407	57.7			1513	55.8			1127	53.7		
≥ College	513	19.0			651	26.7			873	32.2			742	35.4		
Employed (%)	872	32.3			968	39.6			1192	43.8			1064	50.6		
Health insurance (%)	2552	94.4			2336	95.6			2609	95.8			2012	95.6		
Geographic region (%)																
Northeast	444	16.4			335	13.7			374	13.7			297	14.1		
Midwest	747	27.6			721	29.6			732	26.9			565	26.8		
South	1020	37.7			914	37.5			1083	39.8			836	39.7		
West	495	18.3			470	19.3			532	19.6			407	19.3		
Childhood abuse (%)	218	8.1			181	7.5			155	5.7			125	6.0		
**Physical Health**																
Diabetes (%)	631	23.4			437	17.9			411	15.1			273	13.0		
Hypertension (%)	1592	58.9			1328	54.4			1387	51.0			1034	49.1		
Stroke (%)	211	7.8			139	5.7			137	5.0			88	4.2		
Cancer (%)	379	14.1			332	13.6			383	14.1			264	12.6		
Heart Disease (%)	665	24.6			496	20.3			527	19.4			339	16.1		
Lung Disease (%)	279	10.3			215	8.8			182	6.7			96	4.6		
Arthritis (%)	1737	64.2			1417	58.0			1511	55.6			1101	52.4		
Overweight/Obesity (%)	2008	75.2			1722	71.2			1929	71.6			1469	70.6		
Physical limitations (%)	808	29.9			487	19.9			371	13.6			236	11.2		
Cognitive impairment (%)	554	20.7			316	13.0			275	10.2			190	9.1		
Chronic pain (%)	1136	42.0			826	33.8			834	30.6			527	25.0		
Self-rated health (range: 1-5)			2.9	1.0			3.3	1.0			3.5	1.0			3.7	0.9
**Health Behaviors**																
Heavy drinking (%)	174	7.9			141	7.2			165	7.5			126	7.4		
Smoking (%)	400	14.9			325	13.4			278	10.3			194	9.3		
Frequent physical activity (%)	1780	65.9			1858	76.1			2210	81.2			1802	85.6		
Sleep problems (%)	780	50.4			562	41.0			539	37.0			325	32.3		
Religious service attendance (%)																
Never	768	28.4			569	23.3			600	22.1			423	20.1		
<1x/week	891	33.0			838	34.3			807	29.7			655	31.1		
≥1x/week	1045	38.7			1034	42.4			1314	48.3			1027	48.8		
**Psychological Well-Being**																
Positive affect (range: 1-5)			3.1	0.8			3.5	0.6			3.8	0.6			4.1	0.6
Life satisfaction (range: 1-7)			4.4	1.5			5.1	1.3			5.4	1.3			5.7	1.3
Optimism (range: 1-6)			3.9	0.9			4.4	0.9			4.8	0.9			5.1	0.8
Purpose in life (range: 1-6)			3.5	0.5			4.4	0.2			5.1	0.2			5.8	0.2
Mastery (range: 1-6)			4.3	1.1			4.7	1.0			5.1	1.0			5.3	0.9
Health mastery (range: 1-10)			6.5	2.5			7.3	2.2			7.8	1.9			8.3	1.8
Financial mastery (range: 1-10)			6.6	2.9			7.2	2.5			7.8	2.2			8.2	2.1
**Psychological Distress**																
Depression (%)	676	25.0			279	11.4			202	7.4			106	5.0		
Depressive symptoms (range: 0-8)			2.1	2.3			1.2	1.8			0.9	1.4			0.6	1.2
Hopelessness (range: 1-6)			3.2	1.3			2.4	1.1			1.9	1.0			1.6	0.8
Negative affect (range: 1-5)			2.0	0.7			1.7	0.6			1.5	0.5			1.4	0.4
Perceived constraints (range: 1-6)			2.8	1.2			2.2	1.1			1.8	0.9			1.5	0.8
**Social Factors**																
Loneliness (range: 1-3)			1.7	0.6			1.5	0.5			1.3	0.4			1.2	0.4
Not living with spouse/partner (%)		959	36.6			720	30.1			681	25.6			508	24.6	
Contact children <1x/week (%)		742	28.1			578	24.2			620	23.3			471	22.8	
Contact other family <1x/week (%)		1325	50.0			1145	47.7			1304	48.6			933	44.9	
Contact friends <1x/week (%)		1151	43.1			830	34.4			873	32.3			516	24.8	
**Personality**																
Openness (range: 1-4)	2.7	0.5			2.9	0.5			3.1	0.5			3.2	0.5		
Conscientiousness (range: 1-4)	3.1	0.5			3.3	0.4			3.5	0.4			3.6	0.3		
Extroversion (range: 1-4)	2.9	0.6			3.2	0.5			3.3	0.5			3.5	0.4		
Agreeableness (range: 1-4)	3.3	0.5			3.5	0.4			3.6	0.4			3.7	0.4		
Neuroticism (range: 1-4)	2.3	0.6			2.1	0.6			1.9	0.5			1.8	0.5		

^a^ This table was created based on non-imputed data. This is why Table 1**‘**s sample size is smaller than the multiply imputed estimates in the other tables.

^b^ All of these variables were used as covariates, and assessed in the pre-baseline wave (t_0_; 2006/2008).

^c^ The percentages in some sections may not add up to 100% due to rounding.

Over the 4-year follow-up period participants in the highest (versus lowest) purpose quartile had 46% reduced risk of mortality (95% CI [0.44, 0.66]; Table 2), 23% reduced risk of stroke (95% CI [0.62, 0.95]), 17% reduced risk of lung disease (95% CI [0.70, 0.98]), 28% reduced risk of physical functioning limitations (95% CI [0.64, 0.81]), and 16% reduced risk of cognitive impairment (95% CI [0.74, 0.96]), conditional on pre-baseline purpose. They also had fewer chronic conditions (β = −0.08, 95% CI [−0.12, −0.04]) and higher self-rated health (β = 0.17, 95% CI [0.10, 0.23]). However, there was little or no evidence of association between purpose and a range of other physical health outcomes including: diabetes, hypertension, cancer, heart disease, arthritis, overweight/obesity, or chronic pain.

**Table 2.** Sense of Purpose in Life and Subsequent Health and Well-Being (Health and Retirement Study [HRS]: N = 12,998).^a,b,c,d^

Participant characteristics	Purpose in life						
	Quartile 1 (*N* = 3,335)	Quartile 2 (*N* = 3,670)		Quartile 3 (*N* = 2,750)		Quartile 4 (*N* = 3,243)	
	(Reference)	RR/OR/β	95% CI	RR/OR/β	95% CI	RR/OR/β	95% CI
**Physical Health**							
All-cause mortality	1.00	0.81	0.71, 0.94**	0.74	0.62, 0.89***	0.54	0.44, 0.66***
Number of chronic conditions	0.00	−0.05	−0.08, −0.01**	−0.07	−0.10, −0.03***	−0.08	−0.12, −0.04***
Diabetes	1.00	1.01	0.92, 1.10	0.95	0.85, 1.07	0.97	0.87, 1.09
Hypertension	1.00	0.99	0.93, 1.05	1.00	0.94, 1.08	1.01	0.94, 1.09
Stroke	1.00	0.93	0.81, 1.07	0.79	0.66, 0.94*	0.77	0.62, 0.95*
Cancer	1.00	0.98	0.88, 1.10	1.00	0.86, 1.15	0.95	0.82, 1.11
Heart disease	1.00	0.96	0.88, 1.05	1.00	0.91, 1.11	0.94	0.84, 1.05
Lung disease	1.00	0.92	0.80, 1.05	0.85	0.72, 1.00*	0.83	0.70, 0.98*
Arthritis	1.00	0.98	0.92, 1.04	0.98	0.92, 1.05	0.98	0.91, 1.06
Overweight/obesity	1.00	1.00	0.94, 1.07	0.98	0.91, 1.05	0.97	0.90, 1.04
Physical limitations	1.00	0.89	0.82, 0.97**	0.80	0.72, 0.89***	0.72	0.64, 0.81***
Cognitive impairment	1.00	0.95	0.86, 1.05	0.92	0.82, 1.02	0.84	0.74, 0.96*
Chronic pain	1.00	1.00	0.93, 1.08	0.98	0.88, 1.10	0.94	0.85, 1.05
Self-rated health	0.00	0.09	0.04, 0.14***	0.13	0.07, 0.19***	0.17	0.10, 0.23***
**Health Behaviors**							
Heavy drinking	1.00	1.02	0.80, 1.31	0.99	0.73,1.33	1.03	0.76, 1.41
Current smoking	1.00	1.09	0.82, 1.44	0.98	0.66, 1.45	1.18	0.78, 1.79
Frequent physical activity	1.00	1.10	1.02, 1.18**	1.15	1.05, 1.25**	1.15	1.05, 1.25**
Sleep problems	1.00	0.95	0.88, 1.03	0.92	0.83, 1.02	0.87	0.77, 0.99 *
**Psychological Well-being**							
Positive affect	0.00	0.25	0.20, 0.29***	0.38	0.33, 0.43***	0.59	0.53, 0.65***
Life satisfaction	0.00	0.15	0.10, 0.20***	0.21	0.15, 0.27***	0.31	0.24, 0.38***
Optimism	0.00	0.17	0.13, 0.21***	0.31	0.24, 0.37***	0.41	0.35, 0.47***
Purpose in life	0.00	0.38	0.34, 0.42***	0.63	0.58, 0.68***	0.92	0.86, 0.98***
Mastery	0.00	0.19	0.14, 0.25***	0.31	0.24, 0.38***	0.44	0.36, 0.53***
Health mastery	0.00	0.15	0.07, 0.22**	0.23	0.15, 0.31***	0.32	0.22, 0.42***
Financial mastery	0.00	0.15	0.10, 0.21***	0.22	0.15, 0.28***	0.32	0.25, 0.40***
**Psychological Distress**							
Depression	1.00	0.85	0.75, 0.97*	0.68	0.57, 0.82***	0.57	0.46, 0.69***
Depressive symptoms	0.00	−0.17	−0.22, −0.12***	−0.24	−0.30, −0.19***	−0.27	−0.32, −0.21***
Hopelessness	0.00	−0.24	−0.31, −0.18***	−0.34	−0.43, −0.25***	−0.45	−0.56, −0.35***
Negative affect	0.00	−0.13	−0.19, −0.07***	−0.20	−0.28, −0.12***	−0.30	−0.39, −0.21***
Perceived constraints	0.00	−0.19	−0.26, −0.12***	−0.31	−0.40, −0.22***	−0.41	−0.51, −0.31***
**Social Factors**							
Loneliness	0.00	−0.17	−0.22, −0.12***	−0.23	−0.31, −0.16***	−0.35	−0.41, −0.29***
Not living with spouse/partner	1.00	1.00	0.92, 1.08	0.97	0.89, 1.07	0.93	0.84, 1.02
Contact children <1x/week	1.00	0.97	0.87, 1.09	0.91	0.81, 1.03	0.93	0.82, 1.07
Contact other family <1x/week	1.00	0.98	0.90, 1.06	0.99	0.89, 1.11	0.97	0.86, 1.08
Contact friends <1x/week	1.00	0.89	0.82, 0.97*	0.86	0.76, 0.96**	0.80	0.71, 0.90***

Abbreviations: CI, confidence interval; OR, odds ratio; RR, risk ratio.

* p < 0.05 before Bonferroni correction; **p < 0.01 before Bonferroni correction; ***p < 0.05 after Bonferroni correction (the p-value cutoff for Bonferroni correction is p = 0.05/35 outcomes = p < 0.001).

^a^ If the reference value is “1,” the effect estimate is OR or RR; if the reference value is “0”, the effect estimate is β.

^b^ The analytic sample was restricted to those who had participated in the baseline wave (t_1_;2010 or 2012). Multiple imputation was performed to impute missing data on the exposure, covariates, and outcomes. All models controlled for pre-baseline sociodemographic characteristics (age, sex, race/ethnicity, marital status, annual household income, total wealth, level of education, employment status, health insurance, geographic region), pre-baseline childhood abuse, pre-baseline religious service attendance, pre-baseline values of the outcome variables (diabetes, hypertension, stroke, cancer, heart disease, lung disease, arthritis, overweight/obesity, physical functioning limitations, cognitive impairment, chronic pain, self-rated health, binge drinking, current smoking status, physical activity, sleep problems, positive affect, life satisfaction, optimism, purpose in life, mastery, health mastery, financial mastery, depressive symptoms, hopelessness, negative affect, perceived constraints, loneliness, living with spouse/partner, contact children <1x/week, contact other family <1x/week, contact friends <1x/week), personality factors (openness, conscientiousness, extroversion, agreeableness, neuroticism) and the pre-baseline value of the exposure. These variables were controlled for in the pre-baseline wave (in t_0_; 2006 or 2008).

^c^ We used an outcome-wide analytic approach, and ran a separate model for each outcome. We also ran a different type of model depending on the nature of the outcome: 1) for each binary outcome with a prevalence of ≥10%, we ran a generalized linear model with a log link and Poisson distribution to estimate a R.R.; 2) for each binary outcome with a prevalence of <10%, we ran a logistic regression model to estimate an OR; and 3) for each continuous outcome, we ran a linear regression model to estimate a β.

^d^ All continuous outcomes were standardized (mean = 0; standard deviation = 1), and β was the standardized effect size.

When considering health behaviors, participants in the highest (versus lowest) purpose quartile had 15% increased likelihood of subsequent engagement in frequent physical activity (95% CI [1.05, 1.25]) and 13% reduced risk of sleep problems (95% CI [0.77, 0.99]), conditional on pre-baseline purpose. However, purpose was not substantially associated with either heavy drinking or smoking.

Additionally, purpose was associated with all psychological well-being and psychological distress factors. For example, those in the highest (versus lowest) purpose quartile subsequently reported higher positive affect (β = 0.59, 95% CI [0.53, 0.65]) and optimism (β = 0.41, 95% CI [0.35, 0.47]), as well as a lower sense of hopelessness (β = −0.45, 95% CI [−0.56, −0.35]) conditional on pre-baseline purpose, also had a 43% (95% CI [0.46, 0.69]) reduced risk of depression.

Finally, purpose was associated with some social factors. Those in the highest (versus lowest) purpose quartile had lower loneliness (β = −0.35, 95% CI [−0.41, −0.29]) and a 20% lower likelihood of infrequent contact with friends (95% CI = 0.71, 0.90), conditional on pre-baseline purpose. However, there was little or no evidence of associations between purpose and other social factors (living with a spouse/partner, contact with: children, other family).

### Additional analyses

We also conducted 4 additional analyses. First, E-value analyses suggested that several associations we observed were at least moderately robust to unmeasured confounding (Table 3) For example, an unmeasured confounder associated with both purpose and stroke by risk ratios of 1.93, each, above and beyond the large array of potential confounders already adjusted for, could explain away the association, but weaker confounding could not; to shift the confidence interval to include the null, an unmeasured confounder associated with both purpose and stroke by risk ratios of 1.29 each could suffice, but weaker confounding could not. Second, conventionally-adjusted covariate models showed estimates that were stronger than the fully-adjusted models and in line with past research (Supplementary Table 2). Third, when reanalyzing the fully-adjusted models after removing anyone with history of a given condition at baseline, estimates were generally stronger (Supplementary Table 2). Fourth, complete-cases analyses provided similar results to the results in the main analyses (Supplementary Table 3).

**Table 3.** Robustness to Unmeasured Confounding (E-Values) for the Associations Between Sense of Purpose in Life (4th Quartile vs. 1st Quartile) and Subsequent Health and Well-Being (N = 12,998).^a, b, c^

	Effect estimate^b^	Confidence interval limit^c^
**Physical Health**		
All-cause mortality	3.13	2.41
Number of chronic conditions	1.36	1.23
Diabetes	1.19	1.00
Hypertension	1.11	1.00
Stroke	1.93	1.29
Cancer	1.28	1.00
Heart disease	1.33	1.00
Lung disease	1.71	1.16
Arthritis	1.17	1.00
Overweight/obesity	1.21	1.00
Physical limitations	2.13	1.77
Cognitive impairment	1.66	1.24
Chronic pain	1.31	1.00
Self-rated health	1.60	1.43
**Health Behaviors**		
Heavy drinking	1.22	1.00
Current smoking	1.64	1.00
Frequent physical activity	1.56	1.29
Sleep problems	1.55	1.11
**Psychological Well-being**		
Positive affect	2.81	2.63
Life satisfaction	1.98	1.80
Optimism	2.26	2.09
Purpose in life	4.03	3.80
Mastery	2.36	2.14
Health mastery	2.01	1.77
Financial mastery	2.02	1.83
**Psychological Distress**		
Depression	2.93	2.25
Depressive symptoms	1.87	1.73
Hopelessness	2.39	2.13
Negative affect	1.95	1.74
Perceived constraints	2.26	2.03
**Social Factors**		
Loneliness	2.10	1.93
Not living with spouse/partner	1.37	1.00
Contact children <1x/week	1.35	1.00
Contact other family <1x/week	1.23	1.00
Contact friends <1x/week	1.83	1.47

^a^ See VanderWeele and Ding (2017)^27^ for the formula for calculating E-values.

^b^ The E-values for effect estimates are the minimum strength of association on the risk ratio scale that an unmeasured confounder would need to have with both the exposure and the outcome to fully explain away the observed association between the exposure and outcome, conditional on the measured covariates.

^c^ The E-values for the limit of the 95% confidence interval (CI) closest to the null denote the minimum strength of association on the risk ratio scale that an unmeasured confounder would need to have with both the exposure and the outcome to shift the confidence interval to include the null value, conditional on the measured covariates.

### Discussion

#### Summary of Findings

In a prospective and nationally representative sample of U.S. adults aged >50, we observed that people in the highest (versus lowest) purpose quartile, even conditional on pre-baseline purpose had better subsequent: physical health (e.g., reduced risk of: stroke, lung disease, mortality, physical functioning limitations, cognitive impairment; lower number of chronic conditions; and higher self-rated health), behavioral health (e.g., higher physical activity and reduced sleep problems), and psychosocial health (e.g., higher life satisfaction, lower negative affect, and more frequent contact with friends) over the 4-year follow-up period. Our results were maintained after controlling for a robust array of potential confounders including sociodemographic, physical health, behavioral, psychological, and social factors—as well control for purpose, and all outcomes, in the pre-baseline wave. Importantly, we also observed that purpose was not associated with a broad range of other physical- (e.g., cancer, diabetes), behavioral- (e.g., smoking), and social-health (e.g., contact with children) outcomes.

#### Results in the context of past research

By controlling for purpose in the pre-baseline wave, we evaluated *changes in* purpose (conditional on the past), and this “excludes” the potential accumulating effects that past purpose has on health over the life course. From a public health or intervention perspective, this is the analysis that is of more relevance. Our results build upon and converge with past work that has evaluated associations between the “prevalence” of purpose with health and well-being outcomes. For example, we observed that higher purpose was associated with better psychosocial health outcomes and health behaviors (e.g., higher physical activity and reduced sleep problems),^23,24,51,52^ as well as reduced risk of disease (e.g., stroke),^33,53^ and mortality.^31^ However, some of our results diverged from some past prevalence of purpose studies and the underlying reasons may stem from a variety of sources including differences in: (a) measurement and specific operationalization of the outcome, (b) measurement of the exposure, (c) sample, (d) covariate control, and (e) control for pre-baseline purpose. However, when controlling for only conventional covariates in secondary analyses (Supplementary Table 2), many of these initially diverging results then in fact converged with past results, suggesting that modeling is a core reason for potential discrepancies.

We also observed that purpose was not associated with a range of other outcomes (e.g. diabetes, hypertension, cancer, heart disease, arthritis, overweight/obesity, chronic pain, heavy drinking, and smoking). The underlying reasons for associations with some outcomes but not others are unclear; thus, further examination of mechanisms and other explanatory factors is important. Of note, we were only able to consider 4 years of follow-up data and this may not be enough time for a psychological variable to exert cumulative effects on chronic conditions. Further, it remains unclear why purpose was associated with some, but not other, health behaviors. One potential hypothesis is that some subgroups of people with high purpose may cope with the stress of striving toward an overarching goal by engaging in unhealthy behaviors if maintaining health is not a core element of their purpose (e.g., people engaging in unhealthy eating or other unhealthy behaviors to cope with the high stress invoked by pursuing their purpose), while other subgroups may abstain from unhealthy behaviors because it hinders them from achieving an overarching goal where it is important to maintain health (e.g., grandparents wanting to remain healthy and see their grandchildren graduate college). Thus, such possible heterogenous effects, may cancel each other out. Our results could have been influenced (moderated) by numerous other factors such as age, socioeconomic status, employment/retirement status, content of one’s purpose, cohort effects. Thus, future work should formally evaluate these potential moderators of purpose and health/well-being associations.^54^

#### Mechanisms

With regard to mechanisms, past research has documented that higher purpose is associated with increased physical activity and decreased sleep problems.^20,23,24,26,51^ This association may be explained by the fact that people with higher purpose differ on a number of processes including enhanced ability to emotionally recover from negative stimuli,^55,56^ increased ability to handle daily stressors,^57^ decreased impulsivity,^58^ enhanced self-efficacy,^52^ and decreased neural conflict when making healthy decisions.^59^ Future research should expand the range of health behaviors considered, and use more objective and precise instruments to assess each behavior. Past studies have also shown that higher purpose is associated with increased use of preventive healthcare use,^25^ and this may be another potential pathway to health. In our results, we observed that higher purpose was associated with higher psychological well-being, lower psychological distress, and higher social well-being. These psychosocial factors in turn, have been associated with enhanced health and reduced risk of mortality,^2^ thereby serving as potential mechanisms. Other work evaluating potential biological pathways show that those with higher purpose display healthier regulation of physiological systems including healthier lipid profiles and reduced allostatic load.^27-29^ Thus, the purpose-health association may be explained in part by a direct effect on biological function.

### Limitations and Strengths

Self-report bias is a possibility as all outcomes were self-reported. However, study participants were unaware of this study’s hypotheses when completing the HRS survey and purpose was reported pre-baseline prior to the assessment of outcomes. Future studies could re-evaluate these findings using objectively assessed physical health and health behavior outcomes, beyond mortality, to address this limitation. Four years of follow-up data were available and may not be long enough for a psychological factor to exert influence on chronic diseases. Thus, future research could evaluate these associations with datasets with longer follow-up times. Confounding by unmeasured third variables is a limitation. However, the prospective nature of the data, and robust covariate control, helps mitigate these potential concerns; further E-value analyses that assessed robustness to unmeasured confounding suggested that several of our observed associations were at least moderately robust to potential unmeasured confounding. Our study also has considerable strengths including the use of a large, diverse, prospective, and nationally representative sample of U.S. adults aged over 50. We also adjusted for pre-baseline values of the exposures, covariates, and outcomes, allowing us to evaluate “incident exposure” rather than “prevalent exposure,” which provides stronger evidence of causality.^46^ Another advantage of this approach is that from a broader meta-science perspective, it is difficult to publish null results; but by examining many associations simultaneously it is possible to provide evidence for the outcomes that purpose appears to change, and also for those that it does not.^36^

## Conclusions

As the number of older adults in our society rapidly increases, comprehensive and multidisciplinary efforts will be needed to meet the unique demands of this growing population, including policy changes and intervention strategies designed to promote good physical, behavioral, psychological, and social health. Early randomized controlled trials, ranging from volunteering to group cognitive behavioral therapy, have explored whether a sense of purpose can potentially be altered, but further work is needed to continue documenting the effectiveness of these interventions.^4,16-19^ Replication of the present findings is also needed to not only document the impact of purpose in life on multiple health outcomes assessed longitudinally, but also to bolster evidence for further development of purpose interventions. Recognizing the need for additional work in these multiple areas could pave the way for wider public policies and practices to promote purpose in life as a novel way of improving well-being and health among our rapidly aging population.

**So What?**

**What is already known on this topic?**

Growing evidence indicates that a higher sense of *purpose* in life (*purpose*) is associated with reduced risk of chronic diseases and mortality.

**What does this article add?**

However, epidemiological studies have not evaluated if change in *purpose* is associated with subsequent health and well-being outcomes. We evaluated if positive change in purpose was associated with better outcomes on 35 indicators of physical health, health behaviors, and psychosocial well-being over time.

**What are the implications for health promotion practice or research?**

With further research, these results suggest that purpose in life might be a valuable target for innovative policy and intervention work aimed at improving health and well-being.

## Supplemental Material

Supplemental Material, sj-pdf-1-ahp-10.1177_08901171211038545 - Sense of Purpose in Life and Subsequent Physical, Behavioral, and Psychosocial Health: An Outcome-Wide Approach Click here for additional data file. Supplemental Material, sj-pdf-1-ahp-10.1177_08901171211038545 for Sense of Purpose in Life and Subsequent Physical, Behavioral, and Psychosocial Health: An Outcome-Wide Approach by Eric S. Kim, Ying Chen, Julia S. Nakamura, Carol D. Ryff and Tyler J. VanderWeele in American Journal of Health Promotion

## References

[1] Suzman R Beard JR Boerma T Chatterji S . Health in an ageing world—what do we know? Lancet. 2015;385(9967):484–486. doi:10.1016/S0140-6736(14)61597-X 25468156 10.1016/S0140-6736(14)61597-X

[2] Kubzansky LD Huffman JC Boehm JK , et al. Positive psychological well-being and cardiovascular health promotion: JACC health promotion series. J Am Coll Cardiol. 2018;72(12):1382–1396. doi:10.1016/j.jacc.2018.07.042 30213332 10.1016/j.jacc.2018.07.042 PMC6289282

[3] VanderWeele TJ . On the promotion of human flourishing. Proc Natl Acad Sci U S A. 2017;114(31):8148–8156. doi:10.1073/pnas.1702996114 28705870 10.1073/pnas.1702996114 PMC5547610

[4] Ryff CD . Psychological well-being revisited: advances in the science and practice of eudaimonia. Psychother Psychosom. 2014;83(1):10–28. doi:10.1159/000353263 24281296 10.1159/000353263 PMC4241300

[5] VanderWeele TJ Chen Y Long K Kim ES Trudel-Fitzgerald C Kubzansky LD . Positive epidemiology? Epidemiology. 2020;31(2):189–193. doi:10.1097/EDE.0000000000001147 31809344 10.1097/EDE.0000000000001147

[6] Kim ES Tkatch R Martin D MacLeod S Sandy L Yeh C . Resilient aging: psychological well-being and social well-being as targets for the promotion of healthy aging. Gerontol Geriatr Med. 2021;7:23337214211002951. doi:10.1177/23337214211002951 10.1177/23337214211002951 PMC7995285 33816707

[7] Levine GN Cohen BE Commodore-Mensah Y , et al. Psychological health, well-being, and the mind-heart-body connection: a scientific statement from the American Heart Association. Circulation. 2021;143(10):e763–e783. doi:10.1161/cir.0000000000000947 33486973 10.1161/CIR.0000000000000947

[8] Steger MF Frazier P Oishi S Kaler M . The meaning in life questionnaire: assessing the presence of and search for meaning in life. J Couns Psychol. 2006;53(1):80–93. doi:10.1037/0022-0167.53.1.80

[9] McKnight PE Kashdan TB . Purpose in life as a system that creates and sustains health and well-being: an integrative, testable theory. Rev Gen Psychol. 2009;13(3):242–251. doi:10.1037/a0017152

[10] Frankl VE . Man’s Search for Meaning. 1st ed. Beacon Press; 2006.

[11] George LS Park CL . The multidimensional existential meaning scale: a tripartite approach to measuring meaning in life. J Posit Psychol. 2017;12(6):613–627. doi:10.1080/17439760.2016.1209546

[12] Heintzelman SJ King LA . Life is pretty meaningful. Am Psychol. 2014;69(6):561–574. doi:10.1037/a0035049 24490907 10.1037/a0035049

[13] Chen Y Kim ES Shields AE VanderWeele TJ . Antecedents of purpose in life: evidence from a lagged exposure-wide analysis. In: Rodriguez-Blazquez C , ed. Cogent Psychol. 2020;7(1):1825043. doi:10.1080/23311908.2020.1825043 33072817 10.1080/23311908.2020.1825043 PMC7560975

[14] Chen Y Kim ES Koh HK Frazier AL VanderWeele TJ . Sense of mission and subsequent health and well-being among young adults: an outcome-wide analysis. Am J Epidemiol. 2019;188(4):664–673. doi:10.1093/aje/kwz009 30649174 10.1093/aje/kwz009 PMC6438813

[15] Weston SJ Lewis NA Hill PL . Building sense of purpose in older adulthood: examining the role of supportive relationships. J Posit Psychol. 2021;16(3):398–406. doi:10.1080/17439760.2020.1725607

[16] Breitbart W Rosenfeld B Pessin H Applebaum A Kulikowski J Lichtenthal WG . Meaning-centered group psychotherapy: an effective intervention for improving psychological well-being in patients with advanced cancer. J Clin Oncol. 2015;33(7):749–754. doi:10.1200/JCO.2014.57.2198 25646186 10.1200/JCO.2014.57.2198 PMC4334778

[17] Klein N . Prosocial behavior increases perceptions of meaning in life. J Posit Psychol. 2017;12(4):354–361. doi:10.1080/17439760.2016.1209541

[18] Friedman EM Ruini C Foy CR Jaros L Love G Ryff CD . Lighten UP! A community-based group intervention to promote eudaimonic well-being in older adults: a multi-site replication with 6 month follow-up. Clin Gerontol. 2019;42(4):387–397. doi:10.1080/07317115.2019.1574944 30767628 10.1080/07317115.2019.1574944 PMC6715420

[19] Reeves SL Henderson MD Cohen GL Steingut RR Hirschi Q Yeager DS . Psychological affordances help explain where a self-transcendent purpose intervention improves performance. J Pers Soc Psychol. 2021;120(1):1–15. doi:10.1037/pspa0000246 32673046 10.1037/pspa0000246 PMC7790870

[20] Kim ES Shiba K Boehm JK Kubzansky LD . Sense of purpose in life and five health behaviors in older adults. Prev Med. 2020;139:106172. doi:10.1016/j.ypmed.2020.106172 32593729 10.1016/j.ypmed.2020.106172 PMC7494628

[21] Kim ES Ryff C Hassett A Brummett C Yeh C Strecher V . Sense of purpose in life and likelihood of future illicit drug use or prescription medication misuse. Psychosom Med. 2020;82(7):715–721. doi:10.1097/PSY.0000000000000842 32697442 10.1097/PSY.0000000000000842 PMC7484217

[22] Hill PL Edmonds GW Hampson SE . A purposeful lifestyle is a healthful lifestyle: linking sense of purpose to self-rated health through multiple health behaviors. J Health Psychol. 2019;24(10):1392–1400. doi:10.1177/1359105317708251 28810459 10.1177/1359105317708251 PMC5665713

[23] Turner AD Smith CE Ong JC . Is purpose in life associated with less sleep disturbance in older adults? Sleep Sci Pract. 2017;1(1):1. doi:10.1186/s41606-017-0015-6

[24] Kim ES Hershner SD Strecher VJ . Purpose in life and incidence of sleep disturbances. J Behav Med. 2015;38(3):590–597. doi:10.1007/s10865-015-9635-4 25822118 10.1007/s10865-015-9635-4

[25] Kim ES Strecher VJ Ryff CD . Purpose in life and use of preventive health care services. Proc Natl Acad Sci U S A. 2014;111(46):16331–16336. doi:10.1073/pnas.1414826111 25368165 10.1073/pnas.1414826111 PMC4246300

[26] Yemiscigil A Vlaev I . The bidirectional relationship between sense of purpose in life and physical activity: a longitudinal study [published online April 23, 2021]. J Behav Med. 2021. doi:10.1007/s10865-021-00220-2 10.1007/s10865-021-00220-2 33891209

[27] Hafez D Heisler M Choi H Ankuda CK Winkelman T Kullgren JT . Association between purpose in life and glucose control among older adults. Ann Behav Med. 2018;52(4):309–318. doi:10.1093/abm/kax012 30084896 10.1093/abm/kax012 PMC6082637

[28] Zilioli S Slatcher RB Ong AD Gruenewald TL . Purpose in life predicts allostatic load ten years later. J Psychosom Res. 2015;79(5):451–457. doi:10.1016/j.jpsychores.2015.09.013 26526322 10.1016/j.jpsychores.2015.09.013 PMC4684637

[29] Steptoe A Fancourt D . Leading a meaningful life at older ages and its relationship with social engagement, prosperity, health, biology, and time use. Proc Natl Acad Sci U S A. 2019;116(4):1207–1212. doi:10.1073/pnas.1814723116 30617082 10.1073/pnas.1814723116 PMC6347683

[30] Kim ES Kawachi I Chen Y Kubzansky LD . Association between purpose in life and objective measures of physical function in older adults. JAMA Psychiatry. 2017;74(10):1039–1045. doi:10.1001/jamapsychiatry.2017.2145 28813554 10.1001/jamapsychiatry.2017.2145 PMC5710461

[31] Cohen R Bavishi C Rozanski A . Purpose in life and its relationship to all-cause mortality and cardiovascular events: a meta-analysis. Psychosom Med. 2016;78(2):122–133. doi:10.1097/PSY.0000000000000274 26630073 10.1097/PSY.0000000000000274

[32] Lewis NA Turiano NA Payne BR Hill PL . Purpose in life and cognitive functioning in adulthood. Neuropsychol Dev Cogn B Aging Neuropsychol Cogn. 2017;24(6):662–671. doi:10.1080/13825585.2016.1251549 27819520 10.1080/13825585.2016.1251549

[33] Yu L Boyle PA Wilson RS Levine SR Schneider JA Bennett DA . Purpose in life and cerebral infarcts in community-dwelling older people. Stroke. 2015;46(4):1071–1076. doi:10.1161/STROKEAHA.114.008010 25791714 10.1161/STROKEAHA.114.008010 PMC4461202

[34] Kim ES Delaney SW Kubzansky LD . Sense of purpose in life and cardiovascular disease: underlying mechanisms and future directions. Curr Cardiol Rep. 2019;21(11):135. doi:10.1007/s11886-019-1222-9 31673815 10.1007/s11886-019-1222-9 PMC10683927

[35] Willroth EC Mroczek DK Hill PL . Maintaining sense of purpose in midlife predicts better physical health. J Psychosom Res. 2021;145:110485. doi:10.1016/j.jpsychores.2021.110485 33845946 10.1016/j.jpsychores.2021.110485 PMC8114231

[36] VanderWeele TJ Mathur MB Chen Y . Outcome-wide longitudinal designs for causal inference: a new template for empirical studies. Stat Sci. 2020;25(3):437–466. doi:10.1214/19-STS728

[37] Ryff CD Singer B . Understanding healthy aging: key components and their integration. In: Bengston VL Gans D Pulney NM Silverstein M , eds. Handbook of Theories of Aging. Springer Publishing Company; 2009:117–144.

[38] Rowe JW Kahn RL . Human aging: usual and successful. Science. 1987;237(4811):143–149. doi:10.1126/science.3299702 3299702 10.1126/science.3299702

[39] Reich JW Zautra AJ Hall JS . Handbook of Adult Resilience. Guilford Press; 2010.

[40] Aldwin CM Igarashi H . Successful, optimal, and resilient aging: a psychosocial perspective. In: Lichtenberg PA Mast BT Carpenter BD Loebach Wetherell J , eds. APA Handbooks in Psychology®. APA Handbook of Clinical Geropsychology, Vol. 1. History and Status of the Field and Perspectives on Aging. American Psychological Association; 2015:331–359.

[41] Depp CA Jeste DV . Definitions and predictors of successful aging: a comprehensive review of larger quantitative studies. Am J Geriatr Psychiatry. 2006;14(1):6–20. doi:10.1097/01.JGP.0000192501.03069.bc 16407577 10.1097/01.JGP.0000192501.03069.bc

[42] Smith J Fisher G Ryan L Clarke P House J Weir D . Psychosocial and lifestyle questionnaire. Survey Research Center University of Michigan Institute for Social Research. 2013. Accessed February 15, 2021. https://hrs.isr.umich.edu/sites/default/files/biblio/HRS2006-2010SAQdoc.pdf

[43] Ryff CD Keyes CLM . The structure of psychological well-being revisited. J Pers Soc Psychol. 1995;69(4):719–727. doi:10.1037/0022-3514.69.4.719 7473027 10.1037//0022-3514.69.4.719

[44] Fisher GG Faul JD Weir DR Wallace RB . Documentation of chronic disease measures in the heath and retirement study (HRS/AHEAD). Survey Research Center University of Michigan Institute for Social Research. 2005. Accessed February 15, 2021. https://hrs.isr.umich.edu/sites/default/files/biblio/dr-009.pdf

[45] Jenkins KR Ofstedal MB Weir D . Documentation of health behaviors and risk factors measured in the health and retirement study (HRS/AHEAD). Survey Research Center University of Michigan Institute for Social Research. 2008. Accessed February 15, 2021. https://hrs.isr.umich.edu/sites/default/files/biblio/dr-010.pdf

[46] VanderWeele TJ Jackson JW Li S . Causal inference and longitudinal data: a case study of religion and mental health. Soc Psychiatry Psychiatr Epidemiol. 2016;51(11):1457–1466. doi:10.1007/s00127-016-1281-9 27631394 10.1007/s00127-016-1281-9

[47] Greenland S Robins JM . Identifiability, exchangeability, and epidemiological confounding. Int J Epidemiol. 1986;15(3):413–419. doi:10.1093/ije/15.3.413 3771081 10.1093/ije/15.3.413

[48] Robins J . Estimation of the time-dependent accelerated failure time model in the presence of confounding factors. Biometrika. 1992;79(2):321–334. doi:10.2307/2336843

[49] VanderWeele TJ Ding P . Sensitivity analysis in observational research: introducing the e-value. Ann Intern Med. 2017;167(4):268–274. doi:10.7326/M16-2607 28693043 10.7326/M16-2607

[50] Sterne JAC White IR Carlin JB , et al. Multiple imputation for missing data in epidemiological and clinical research: potential and pitfalls. BMJ. 2009;338:b2393. doi:10.1136/bmj.b2393 19564179 10.1136/bmj.b2393 PMC2714692

[51] Hooker SA Masters KS . Purpose in life is associated with physical activity measured by accelerometer. J Health Psychol. 2016;21(6):962–971. doi:10.1177/1359105314542822 25104777 10.1177/1359105314542822

[52] Rush CL Hooker SA Ross KM Frers AK Peters JC Masters KS . Brief report: meaning in life is mediated by self-efficacy in the prediction of physical activity. J Health Psychol. 2021;26(5):753–757. doi:10.1177/1359105319828172 30791727 10.1177/1359105319828172

[53] Kim ES Sun JK Park N Peterson C . Purpose in life and reduced incidence of stroke in older adults: the health and retirement study. J Psychosom Res. 2013;74(5):427–432. doi:10.1016/j.jpsychores.2013.01.013 23597331 10.1016/j.jpsychores.2013.01.013

[54] Ryff CD Kim ES . Extending research linking purpose in life to health: the challenges of inequality, the potential of the arts, and the imperative of virtue. In: Burrow AL Hill PL , eds. The Ecology of Purposeful Living Across the Lifespan. Springer; 2020:29–58.

[55] Schaefer SM Morozink Boylan J van Reekum CM , et al. Purpose in life predicts better emotional recovery from negative stimuli. PLoS One. 2013;8(11):e80329. doi:10.1371/journal.pone.0080329 24236176 10.1371/journal.pone.0080329 PMC3827458

[56] Fogelman N Canli T . Purpose in life as a psychosocial resource in healthy aging: an examination of cortisol baseline levels and response to the trier social stress test. NPJ Aging Mech Dis. 2015;1:15006. doi:10.1038/npjamd.2015.6 28721255 10.1038/npjamd.2015.6 PMC5514985

[57] Hill PL Sin NL Turiano NA Burrow AL Almeida DM . Sense of purpose moderates the associations between daily stressors and daily well-being. Ann Behav Med. 2018;52(8):724–729. doi:10.1093/abm/kax039 30010709 10.1093/abm/kax039 PMC6052784

[58] Burrow AL Spreng RN . Waiting with purpose: a reliable but small association between purpose in life and impulsivity. Pers Individ Dif. 2016;90:187–189. doi:10.1016/j.paid.2015.11.010 26640312 10.1016/j.paid.2015.11.010 PMC4668943

[59] Kang Y Strecher VJ Kim ES Falk EB . Purpose in life and conflict-related neural responses during health decision making. Health Psychol. 2019;38(6):545–552. doi:10.1037/hea0000729 31008647 10.1037/hea0000729 PMC7233478

