# Supplementary material for: Sense of Purpose in Life and Subsequent Physical, Behavioral, and Psychosocial Health: An Outcome-Wide Approach
Source: Am J Health Promot. 2021 Aug 18;36(1):137–47. doi: 10.1177/08901171211038545 (PMC8669210; doi:10.1177/08901171211038545)
Supplement: Supplemental Material, sj-pdf-1-ahp-10.1177_08901171211038545 - Sense of Purpose in Life and Subsequent Physical, Behavioral, and Psychosocial Health: An Outcome-Wide Approach [file sj-pdf-1-ahp-10.1177_08901171211038545.pdf]

Sense of Purpose in Life and Subsequent Physical, Behavioral, and Psychosocial Health: An Outcome-Wide Approach

Supplementary Text 1. Assessment of Outcomes

Supplementary Text 2. Proof Illustrating How Controlling for Pre-Baseline Levels of Purpose in Life Can Help Us Evaluate How "Change" in Purpose in Life is Associated with Subsequent Health and Well-Being Outcomes Over Time

Supplementary Table 1. Change in Sense of Purpose in Life from the Pre-Baseline Wave (t_0) to the Baseline Wave (t_1)

Supplementary Table 2. Sense of Purpose in Life and Subsequent Health and Well-being (After Adjustment for Conventional Covariates or All Covariates) (Health and Retirement Study [HRS]: N =12,998)

Supplementary Table 3. Complete-Case Analyses: Sense of Purpose in Life and Subsequent Health and Well-being (Health and Retirement Study [HRS]: N ranged from: 5,531 to 8,352)

Supplementary text 1.

Assessment of outcomes

Reference group

Unless otherwise noted, for all binary outcomes the reference group was the healthiest group.

Physical health

All-cause mortality. Information about death was obtained up to the 2016 questionnaire wave via two methods. First, an exit interview was conducted with next-of-kin. Then, after each wave of data collection, the National Death Index (NDI) was searched for death information. When comparing deaths reported by NDI versus exit interviews, there is a 95.5% match.[1]

Chronic conditions. Participants self-reported whether they were ever told by a healthcare provider that they had (yes/no) the following conditions: 1) diabetes, 2) hypertension, 3) stroke, 4) cancer, 5) heart disease, 6) lung disease, or 7) arthritis. Validity and reliability of self-reported chronic conditions has previously been demonstrated in HRS.[2]

***Overweight/obesity*.** Body mass index (BMI) was derived based on self-reported height and weight, and BMI was calculated as weight/height^2 (kg/m^2). A BMI of ≥25 kg/m^2 was considered as overweight/obese.[3]

Number of chronic conditions. To create a number of chronic conditions score, a summary score was calculated by summing the number of reported conditions (e.g., the 7 chronic conditions and also overweight/obesity; range=0 to 8).

Cognitive function problem. The HRS cognitive function assessment[4,5] was adapted from the modified Telephone Interview for Cognitive Status (TICS-M). The assessment is a 27-point scale that included an immediate and delayed 10-noun free recall test, a serial 7 subtraction test, and a backward count 20 test. This assessment tool has been shown to have high sensitivity and specificity for cognitive impairment in older adults; cutpoints were derived from previous research conducted on cognitive impairment in HRS.[6,7] Respondents scoring 0-11 on the 27-point scale were classified as having "cognitive impairment," while those scoring ≥12 were classified as "normal" (the reference group). More detailed information about the cognitive assessments can be found in HRS reports.[4,5]

Physical functioning limitations. Physical functioning limitations were assessed using items adapted from scales developed by Rosow and Breslau (1966), Nagi (1976), Katz, Ford, Moskowitz, Jackson, and Jaffe (1963), and Lawton and Brody (1969).[8–11] Participants were defined as having physical function limitations if they reported >4 limitations with physical functioning (i.e., walking several blocks, climbing one flight of stairs, pushing or pulling large objects, lifting or carrying 10 pounds, getting up from a chair, reaching or extending arms up, stooping, kneeling, or crouching, sitting for 2 hours) or activities of daily living (i.e., walking across a room, dressing, eating, bathing, getting in/out bed, using the toilet, picking up a dime). Those reporting ≤4 limitations were considered "normal" in the physical function domain and served as the reference group. This criterion was determined by identifying the physical function score where 75% of participants could be considered as having healthy physical function at baseline.

Chronic pain. Chronic pain was assessed by asking respondents (yes/no): "Are you often troubled with pain?" No pain was the reference group.

Self-rated health. Participants were asked, "Would you say your health is excellent, very good, good, fair, or poor?" on a 5-point scale (reverse coded with higher scores indicating higher self-rated health).

Health behaviors

Heavy drinking. Following the National Institute on Alcohol Abuse and Alcoholism guidelines,[12] heavy drinking was defined as >14 for drinks/week for men and >7 drinks/week for women. Alcohol consumption was measured by multiplying the number of days/week that alcohol was consumed x number of drinks/day, which resulted in the number of drinks/week. Participants not in this alcohol consumption range were classified as non-heavy drinkers (the reference group).

Smoking. Current smoking status was assessed by asking participants: "Do you smoke cigarettes now?" The response categories included "yes" or "no" (with "no" smoking as the reference group).

Frequent physical activity. Based on prior research, we created a binary physical activity variable where ≥1x/week of vigorous or moderate exercise was considered frequent physical activity and <1x/week of vigorous or moderate exercise was the reference group.[13] Physical activity was measured by asking participants their frequency of engaging in vigorous (e.g.,

running, swimming, aerobics), moderate (e.g., gardening, dancing, walking at a moderate pace), and light (e.g., vacuuming, laundry) activities over the past 12 months. Response categories included daily, >1x/week, 1x/week, 1-3x/month, hardly ever or never.

Sleep problems. Participants completed the 4-item Jenkins Sleep Questionnaire, a validated and widely used screening instrument for sleep complaints, querying insomnia symptoms.[14] Response categories included "most of the time," "sometimes," and "rarely or never." Having sleep problems was defined as reporting: "most of the time" for any of the three negatively worded items (e.g., "How often do you have trouble falling asleep?") and "rarely or never" to the one positively worded item (i.e., "feel really rested when you wake up in the morning"). Participants were considered unhealthy (i.e., having sleep problems) if they reported one or more sleep problems. The sleep questionnaire was only administered every other wave. Thus, sleep data was imputed for half of the sample. Imputed and complete-case analyses showed similar estimates.

Psychological well-being

Positive affect. Positive affect was measured (in 2006 only) with a 6-item scale originally developed for use in the Midlife in the United States Study.[15–17] The scale assessed how often the participant felt "cheerful," "in good spirits," "extremely happy," "calm and peaceful," "satisfied," and "full of life" over the past 30 days. Response categories ranged from 1 (all of the time) to 5 (none of the time). Responses were reverse scored, so that a higher score indicated higher positive affect. An overall score was derived by averaging responses across all 6 items (α=0.91 in 2006, range=1 to 5). After the 2006 wave, the HRS switched to a more expansive

measure of positive affect based on the Positive and Negative Affect Schedule (PANAS-X).[18] It included the following 13 items: determined, enthusiastic, active, proud, interested, happy, attentive, content, inspired, hopeful, alert, calm, excited. An overall score was derived by averaging responses across all 13 items (α=0.92, range=1 to 5). A limitation of this study is that affect was measured in a different way during only the first wave of the study. However, scores were standardized and both the prior and current measures of affect operate very similarly (e.g., similar correlations with other variables, similar distributions, etc.).

Life satisfaction. Life satisfaction was assessed with the 5-item Satisfaction with Life Scale (e.g., "In most ways my life is close to ideal").[19] The scale has shown excellent psychometric properties in prior work. Response categories ranged from 1 (strongly disagree) to 7 (strongly agree). An overall score was derived by averaging responses across all 5 items, with a higher score indicating higher life satisfaction (α=0.88, range=1 to 7).

Optimism. Optimism was assessed using the Life Orientation Test-Revised (LOT-R). The measure has good discriminant and convergent validity, and good reliability.[20] Using a 6-point Likert scale (from 1 (strongly disagree) to 6 (strongly agree)), participants were asked the degree to which they agreed with six statements such as, "In uncertain times, I usually expect the best." After reverse coding negatively worded items, all items were averaged together to create a composite score, with higher scores indicating higher optimism (α=0.75, range=1 to 6).

Mastery. Mastery was measured with 5-items derived from Lachman and Weaver (1998), and rated on a scale from 1 (strongly disagree) to 6 (strongly agree). The measure has good

discriminant and convergent validity, as well as good reliability.[21] Participants were asked the degree to which they agreed with five statements such as, "I can do just about anything I really set my mind to." All items were averaged together to create a composite score, with higher scores indicating higher mastery (α=0.90, range=1 to 6).

Health mastery. On a 0 to 10 scale where 0 means "no control at all" and 10 means "very much control," participants were asked, "how would you rate the amount of control you have over your health these days?"

Financial mastery. On a 0 to 10 scale where 0 means "no control at all" and 10 means "very much control," participants were asked, "how would you rate the amount of control you have over your financial situation these days?"

Psychological distress

Depressive symptoms and depression. Depressive symptoms over the past week were measured using the 8-item Center for Epidemiologic Studies Depression Scale (CESD)[22] (e.g., "Much of the time during the past week, I felt depressed"), and response options included "yes" or "no" for each item. Following HRS protocol, an overall score was derived ranging from 0 to 8, with a higher score indicating higher depressive symptoms. The scale has been previously validated in the Health and Retirement Study[23] and showed high reliability in this sample (α=0.80). Following prior work,[23] participants with a score of ≥4 were considered as having significant depressive symptoms, or depression. Prior work suggested that the cutoff of 4 would produce

comparable results as the 16 symptoms cutoff when using the full 20-item CESD scale.[23] No depression was the reference group.

Hopelessness. Hopelessness was measured with 4 questionnaire items from two previously validated scales[24,25] (e.g., "I feel it is impossible for me to reach the goals that I would like to strive for", "The future seems hopeless to me and I can't believe that things are changing for the better"). Response categories ranged from 1 (strongly disagree) to 6 (strongly agree). An overall score was created by averaging the responses across all items (α=0.87, range=1 to 6).

Negative affect. Negative affect was measured (in 2006 only) with a 6-item scale originally developed for use in the Midlife in the United States Study.[15–17] The scale assessed how often the participant felt "so depressed that nothing could cheer you up," "hopeless," "restless or fidgety," "that everything was an effort," "worthless," and "nervous" over the past 30 days. Response categories ranged from 1 (all of the time) to 5 (none of the time). Responses were reverse scored, so that a higher score indicated higher negative affect. An overall score was derived by averaging responses across all 6 items (α=0.86 in 2006, range=1 to 5). After the 2006 wave, the HRS switched to a more expansive measure of negative affect based on the Positive and Negative Affect Schedule (PANAS-X).[18] It included the following 12 items: afraid, upset, guilty, scared, frustrated, bored, hostile, jittery, ashamed, nervous, sad, distressed. An overall score was derived by averaging responses across all 12 items (α=0.89, range=1 to 5). A limitation of this study is that affect was measured in a different way during only the first wave of the study. However, scores were standardized and both the prior and current measures of affect operate very similarly (e.g., similar correlations with other variables, similar distributions, etc.).

Perceived constraints. Perceived constraints were assessed with 5 items derived from Lachman and Weaver (1998), and this measure has good discriminant and convergent validity, as well as good reliability.[21] Using a 6-point Likert scale (from 1 (strongly disagree) to 6 (strongly agree)), participants were asked the degree to which they agreed with statements such as, "What happens in my life is often beyond my control." All items were averaged to create an overall score, with higher scores indicating a higher sense of constraints on personal control (α=0.86, range 1-6).

Social factors

Loneliness. Loneliness was measured with three items from the previously validated UCLA Loneliness Scale (i.e., How much of the time do you feel: 1) you lack companionship, 2) left out, and 3) isolated from others).[26] Response categories ranged from 1 (often) to 3 (hardly ever or never). Responses were reverse scored, so that a higher score indicated higher loneliness. An overall score was derived by averaging the responses across the three items (α=0.80, range=1 to 3).

Living with partner/spouse. Participants were asked (yes/no), "Do you have a husband, wife, or partner with whom you live?"

Frequency of contact with: children, other family, and friends. Frequency of contact with children, other family, or friends was each queried separately, but in the same way. For example, participants were asked: "On average, how often do you do each of the following?" 1) "Meet up (include both arranged and chance meetings)," 2) "Speak on the phone," 3) "Write or email." For

each of these 3 categories of questions, HRS respondents had the option of choosing 1 of the following 6 responses: 1) ≥3x/week, 2) 1x-2x/week, 3) 1-2x/month, 4) every few months, 5) 1-2x/year, 6) <1x/year or never.[27] Because contact of any kind (regardless of medium) was the main point of intertest, the highest value on any of the three modes of contact (e.g., meet up, phone, write/email) was taken. In other words, if the respondent did not meet in person very often with the other person but spoke on the phone very often with that person, contact was operationalized as fairly common, given that they speak on the phone very often. A binary frequency of contact variable was created where <1x/week of contact was considered infrequent contact and ≥1x/week contact was considered frequent contact and (with this serving as the reference group).

Other factors

Personality. The "Big-5" personality traits (openness to experience, conscientiousness, extraversion, agreeableness, and neuroticism)[28] were measured using 26 items derived from the Midlife Development Inventory Personality scales (MIDI) and International Personality Item Pool (IPIP). Using existing trait inventories, the goal of MIDI was to create the shortest possible collection of items that measured the Big-Five personality traits with high validity and reliability. In a pilot study conducted among a probability sample of 1,000 adults aged 30-70, items with the highest item-to-total correlations and factor loadings were selected for the MIDI. Forward regressions were then computed to determine the smallest number of items needed to account for more than 90 percent of the total scale variance. As an illustrative example, items on the conscientiousness scale included "organized," "responsible," "hardworking," and "careless." Response categories ranged from 1 (a lot) to 4 (not at all). Responses were reverse scored, so that

a higher score indicated higher indication of a given personality trait. An overall score for each personality trait was derived by averaging responses across all items of a given Big-5 Personality variable.

Supplementary text 2.

Proof illustrating how controlling for pre-baseline levels of purpose in life can help us evaluate how "change" in purpose in life is associated with subsequent health and well-being outcomes over time

Let Y be the outcome in 2014/2016, A_1 the purpose in life exposure in 2010/2012, A_0 the purpose in life exposure in 2006/2008, C the set of covariates in 2006/2008. For a continuous outcome, the regression model is: $E[Y|a_0, a_1, c] = v + b_0a_0 \;\; b_1a_1 + b_2'c$

Let Y_a denote the potential outcome for Y for an individual under an intervention to set A_1 to a. For an individual with baseline purpose in life exposure $A_0=a_0$ and covariates c in 2006/2008, under the no-confounding (and positivity and consistency) and modeling assumptions, a change in purpose in life of d points $A_0=a_0$ to $A_1=a_0+d$ in 2010/2012, rather than maintaining purpose in life of $A_1=a_0$ in 2010/2012, will give rise to an effect (a difference in potential outcomes for Y) of:

$E[Y_{a0+d}| A_0=a_0, c] - E[Y_{a0}| A_0=a_0, c]$

$= E[Y_{a0+d}| A_1=a_0+d, A_0=a_0, c] - E[Y_{a0}| A_1=a_0, A_0=a_0, c]$

$= E[Y| A_1=a_0+d, A_0=a_0, c] - E[Y| A_1=a_0, A_0=a_0, c]$

$= [v + b_0a_0 + b_1(a_0+d) + b_2'c] - [v + b_0a_0 + b_1a_0 + b_2'c]$

$= b_1d$

where the first equality follows by the no-confounding assumption, the second by consistency, and the third by the statistical model.

Supplementary Table 1. Change in Sense of Purpose in Life from the Pre-Baseline Wave (t_0) to the Baseline Wave (t_1) [a.b]

	**Baseline Wave (t_1)**			
	Quartile 1<br>%	Quartile 2<br>%	Quartile 3<br>%	Quartile 4<br>%
**Pre-Baseline Wave (t_0)**				
Quartile 1	57.5	28.3	9.2	5.0
Quartile 2	25.9	38.9	21.5	13.7
Quartile 3	11.6	30.9	30.0	27.5
Quartile 4	4.9	15.1	25.0	55.1

[a]The percent of people in quartile 1, 2, 3, or 4 in the pre-baseline wave ($t_{0;}$ 2006/2008) who (four years later) end up in quartile 1, 2, 3, or 4 in the baseline wave ($t_{1;}$ 2010/2012).
[b]The values in the fourth row (quartile 4) do not add up to 100% because of rounding.

Supplementary Table 2. Sense of Purpose in Life and Subsequent Health and Well-being (After Adjustment for Conventional Covariates or All Covariates) (Health and Retirement Study [HRS]: N = 12,998)[a,b,c]

	Purpose in Life				
	Quartile 1	Conventionally-Adjusted Models Quartile 4[d]		Fully-Adjusted Models Quartile 4[e]	
	(Reference)	RR/OR/β	95% CI	RR/OR/β	95% CI
**Physical Health**					
All-cause mortality	1.00	0.44	0.36, 0.52***	0.54	0.44, 0.66***
Number of chronic conditions	0.00	-0.29	-0.34, -0.24***	-0.08	-0.12, -0.04***
Diabetes[f]	1.00	0.84	0.63, 1.12	0.87	0.65, 1.18
Hypertension[g]	1.00	1.04	0.85, 1.27	1.19	0.93, 1.53
Stroke[h]	1.00	0.57	0.41, 0.78***	0.61	0.41, 0.92*
Cancer[i]	1.00	0.91	0.67, 1.23	0.81	0.54, 1.24
Heart disease[j]	1.00	0.85	0.69, 1.05	0.90	0.70, 1.17
Lung disease[k]	1.00	0.67	0.50, 0.88**	0.91	0.62, 1.32
Arthritis[l]	1.00	0.75	0.62, 0.91**	0.89	0.68, 1.16
Overweight/obesity[m]	1.00	0.88	0.69, 1.13	0.79	0.59, 1.06
Physical limitations[n]	1.00	0.53	0.45, 0.62***	0.67	0.55, 0.81***
Cognitive impairment[o]	1.00	0.77	0.63, 0.94*	0.90	0.70, 1.14
Chronic pain[p]	1.00	0.75	0.65, 0.87***	0.89	0.75, 1.05
Self-rated health	0.00	0.53	0.48, 0.58***	0.17	0.10, 0.23***
**Health Behaviors**					
Heavy drinking	1.00	1.01	0.78, 1.31	1.03	0.76, 1.41
Current smoking	1.00	0.63	0.50, 0.79***	1.18	0.78, 1.79
Frequent physical activity	1.00	1.33	1.25, 1.43***	1.15	1.05, 1.25**
Sleep problems	1.00	0.68	0.62, 0.74***	0.87	0.77, 0.99*
**Psychological Well-being**					
Positive affect	0.00	1.20	1.14, 1.25***	0.59	0.53, 0.65***
Life satisfaction	0.00	0.73	0.68, 0.78***	0.31	0.24, 0.38***
Optimism	0.00	1.00	0.94, 1.07***	0.41	0.35, 0.47***
Purpose in life	0.00	1.51	1.47, 1.55***	0.92	0.86, 0.98***

Mastery	0.00	0.86	0.80, 0.93***	0.44	0.36, 0.53***
Health mastery	0.00	0.63	0.56, 0.70***	0.32	0.22, 0.42***
Financial mastery	0.00	0.63	0.57, 0.69***	0.32	0.25, 0.40***
**Psychological Distress**					
Depression	1.00	0.28	0.23, 0.33***	0.57	0.46, 0.69***
Depressive symptoms	0.00	-0.68	-0.74, -0.62***	-0.27	-0.32, -0.21***
Hopelessness	0.00	-0.98	-1.04, -0.91***	-0.45	-0.56, -0.35***
Negative affect	0.00	-0.78	-0.84, -0.71***	-0.30	-0.39, -0.21***
Perceived constraints	0.00	-0.91	-0.98, -0.84***	-0.41	-0.51, -0.31***
**Social Factors**					
Loneliness	0.00	-0.81	-0.87, -0.75***	-0.35	-0.41, -0.29***
Not living with spouse/partner	1.00	0.88	0.81, 0.96**	0.93	0.84, 1.02
Contact children <1x/week	1.00	0.79	0.72, 0.88***	0.93	0.82, 1.07
Contact other family <1x/week	1.00	0.88	0.81, 0.94***	0.97	0.86, 1.08
Contact friends <1x/week	1.00	0.67	0.62, 0.73***	0.80	0.71, 0.90***

*p<0.05 before Bonferroni correction; **p<0.01 before Bonferroni correction; ***p<0.05 after Bonferroni correction (the p-value cutoff for Bonferroni correction is p=0.05/35 outcomes=p<0.001)).

Abbreviations: CI, confidence interval; OR, odds ratio; RR, risk ratio.

[a] If the reference value is "1," the effect estimate is OR or RR; if the reference value is "0," the effect estimate is β.

[b] An outcome-wide analytic approach was used, and a separate model for each outcome was run. A different type of model was run depending on the nature of the outcome: 1) for each binary outcome with a prevalence of ≥10%, a generalized linear model (with a log link and Poisson distribution) was used to estimate a RR; 2) for each binary outcome with a prevalence of <10%, a logistic regression model was used to estimate an OR; and 3) for each continuous outcome, a linear regression model was used to estimate a β.

[c] All continuous outcomes were standardized (mean=0; standard deviation=1), and β was the standardized effect size.

[d] The analytic sample was restricted to those who had participated in the baseline wave (t_1;2010 or 2012). Multiple imputation was performed to impute missing data on the exposure, covariates, and outcomes. All models controlled for sociodemographic characteristics (age, sex, race/ethnicity, marital status, annual household income, total wealth, level of education). These variables were controlled for in the pre-baseline wave (t_0;in 2006 or 2008).

[e] The analytic sample was restricted to those who had participated in the baseline wave (t_1;2010 or 2012). Multiple imputation was performed to impute missing data on the exposure, covariates, and outcomes. All models controlled for sociodemographic characteristics (age, sex, race/ethnicity, marital status, annual household income, total wealth, level of education, employment status, health insurance, geographic region), pre-baseline childhood abuse, pre-baseline religious service attendance, pre-baseline values of the outcome variables (diabetes, hypertension, stroke, cancer, heart disease, lung disease, arthritis, overweight/obesity, physical

functioning limitations, cognitive impairment, chronic pain, self-rated health, heavy drinking, current smoking status, physical activity, sleep problems, positive affect, life satisfaction, optimism, purpose in life, mastery, health mastery, financial mastery, depressive symptoms, hopelessness, negative affect, perceived constraints, loneliness, living with spouse/partner, contact children <1x/week, contact other family <1x/week, contact friends <1x/week), personality factors (openness, conscientiousness, extraversion, agreeableness, neuroticism) and the pre-baseline value of the exposure. These variables were controlled for in the pre-baseline wave (in t_0;2006 or 2008).

[f]includes only study participants with no history of diabetes (n=10,032).

[g]includes only study participants with no history of hypertension (n=5,145). For this analysis, we did not control for hypertension in wave 1 because the cell size was too small and the analysis did not converge.

[h]includes only study participants with no history of stroke (n=11,916).

[i]includes only study participants with no history of cancer (n=10,849).

[j]includes only study participants with no history of heart disease (n=9,714).

[k]includes only study participants with no history of lung disease (n=11,676).

[l]includes only study participants with no history of arthritis (n=5,024). For this analysis, we did not control for arthritis in wave 1. because the cell size was too small and the analysis did not converge.

[m]includes only study participants who were not overweight/obese (n=3,752).

[n]includes only study participants who did not have physical limitations (n=9,797).

[o]includes only study participants who did not have cognitive impairment (n=10,413).

[p]includes only study participants who did not have chronic pain (n=8,286).

Supplementary Table 3. Complete-Case Analyses: Sense of Purpose in Life and Subsequent Health and Well-being (Health and Retirement Study [HRS]: N ranged from: 5,531 to 8,353)[a,b,c,d]

**Participant Characteristics**	**Purpose in Life**						
	Quartile 1	Quartile 2		Quartile 3		Quartile 4	
	(Reference)	RR/OR/β	95% CI	RR/OR/β	95% CI	RR/OR/β	95% CI
**Physical Health**							
All-cause mortality	1.00	0.85	0.72, 1.00	0.74	0.60, 0.91**	0.57	0.44, 0.74***
Number of chronic conditions	0.00	-0.04	-0.09, 0.00*	-0.07	-0.12, -0.02**	-0.08	-0.13, -0.03**
Diabetes	1.00	1.00	0.88, 1.14	0.99	0.85, 1.15	0.99	0.83, 1.17
Hypertension	1.00	0.98	0.90, 1.06	0.99	0.90, 1.09	0.98	0.88, 1.10
Stroke	1.00	0.97	0.79, 1.18	0.81	0.63, 1.03	0.73	0.55, 0.97*
Cancer	1.00	1.01	0.87, 1.18	1.06	0.89, 1.26	0.98	0.81, 1.19
Heart disease	1.00	0.92	0.81, 1.03	0.95	0.83, 1.09	0.90	0.77, 1.05
Lung disease	1.00	0.93	0.77, 1.13	0.83	0.67, 1.04	0.86	0.67, 1.12
Arthritis	1.00	0.98	0.90, 1.06	0.97	0.89, 1.07	0.99	0.90, 1.11
Overweight/obesity	1.00	1.01	0.93, 1.10	0.98	0.89, 1.07	0.98	0.88, 1.09
Physical limitations	1.00	0.85	0.76, 0.96*	0.74	0.64, 0.86***	0.72	0.61, 0.86***
Cognitive impairment	1.00	0.97	0.84, 1.11	0.89	0.75, 1.06	0.88	0.72, 1.07
Chronic pain	1.00	1.00	0.90, 1.11	0.98	0.86, 1.10	0.98	0.85, 1.12
Self-rated health	0.00	0.08	0.03, 0.14**	0.12	0.06, 0.18***	0.12	0.05, 0.19***
**Health Behaviors**							
Heavy drinking	1.00	1.16	0.82, 1.64	1.13	0.78, 1.65	1.07	0.70, 1.63
Current smoking	1.00	0.88	0.60, 1.27	0.75	0.49, 1.15	1.07	0.66, 1.73
Frequent physical activity	1.00	1.08	0.98, 1.19	1.17	1.06, 1.29**	1.15	1.03, 1.29*
Sleep problems	1.00	1.02	0.91, 1.13	0.94	0.83, 1.06	0.96	0.83, 1.10
**Psychological Well-being**							
Positive affect	0.00	0.24	0.19, 0.30***	0.40	0.33, 0.46***	0.60	0.52, 0.67***
Life satisfaction	0.00	0.18	0.12, 0.24***	0.25	0.18, 0.32***	0.32	0.24, 0.39***
Optimism	0.00	0.17	0.11, 0.23***	0.31	0.24, 0.37***	0.38	0.30, 0.45***

Purpose in life	0.00	0.36	0.31, 0.42***	0.67	0.61, 0.74***	0.95	0.88, 1.02***
Mastery	0.00	0.18	0.12, 0.25***	0.33	0.26, 0.40***	0.47	0.38, 0.55***
Health mastery	0.00	0.14	0.07, 0.21***	0.24	0.16, 0.31***	0.34	0.26, 0.42***
Financial mastery	0.00	0.17	0.11, 0.24***	0.25	0.17, 0.32***	0.36	0.28, 0.45***
**Psychological Distress**							
Depression	1.00	0.80	0.68, 0.94**	0.62	0.50, 0.76***	0.57	0.45, 0.74***
Depressive symptoms	0.00	-0.14	-0.20, -0.09***	-0.24	-0.31, -0.18***	-0.22	-0.29, -0.15***
Hopelessness	0.00	-0.27	-0.33, -0.21***	-0.35	-0.42, -0.28***	-0.42	-0.49, -0.34***
Negative affect	0.00	-0.09	-0.16, -0.03**	-0.18	-0.25, -0.11***	-0.26	-0.34, -0.19***
Perceived constraints	0.00	-0.20	-0.27, -0.14***	-0.34	-0.41, -0.27***	-0.43	-0.50, -0.35***
**Social Factors**							
Loneliness	0.00	-0.19	-0.25, -0.13***	-0.26	-0.33, -0.19***	-0.33	-0.41, -0.25***
Not living with spouse/partner	1.00	1.00	0.89, 1.14	0.95	0.82, 1.09	0.91	0.77, 1.07
Contact children <1x/week	1.00	0.97	0.85, 1.11	0.88	0.75, 1.03	0.91	0.76, 1.08
Contact other family <1x/week	1.00	1.01	0.91, 1.12	1.04	0.92, 1.17	0.95	0.83, 1.09
Contact friends <1x/week	1.00	0.88	0.78, 0.99*	0.85	0.74, 0.97*	0.79	0.68, 0.93**

*p<0.05 before Bonferroni correction; **p<0.01 before Bonferroni correction; ***p<0.05 after Bonferroni correction (the p-value cutoff for Bonferroni correction is p=0.05/35 outcomes=p<0.001).

Abbreviations: CI, confidence interval; OR, odds ratio; RR, risk ratio.

[a]If the reference value is "1," the effect estimate is OR or RR; if the reference value is "0," the effect estimate is β.

[b]The analytic sample was restricted to those who had participated in the baseline wave (t_1;2010 or 2012). All models controlled for pre-baseline sociodemographic characteristics (age, sex, race/ethnicity, marital status, annual household income, total wealth, level of education, employment status, health insurance, geographic region), pre-baseline childhood abuse, pre-baseline religious service attendance, pre-baseline values of the outcome variables (diabetes, hypertension, stroke, cancer, heart disease, lung disease, arthritis, overweight/obesity, physical functioning limitations, cognitive impairment, chronic pain, self-rated health, heavy drinking, current smoking status, physical activity, sleep problems, positive affect, life satisfaction, optimism, purpose in life, mastery, health mastery, financial mastery, depressive symptoms, hopelessness, negative affect, perceived constraints, loneliness, living with spouse/partner, contact children <1x/week, contact other family <1x/week, contact friends <1x/week), personality factors (openness, conscientiousness, extraversion, agreeableness, neuroticism) and the pre-baseline value of the exposure. These variables were controlled for in the pre-baseline wave (in t_0;2006 or 2008).

[c]We used an outcome-wide analytic approach, and ran a separate model for each outcome. We also ran a different type of model depending on the nature of the outcome: 1) for each binary outcome with a prevalence of ≥10%, we ran a generalized linear model with a log link and Poisson distribution to estimate a RR; 2) for each binary outcome with a prevalence of <10%, we ran a logistic regression model to estimate an OR; and 3) for each continuous outcome, we ran a linear regression model to estimate a β.
[d]All continuous outcomes were standardized (mean = 0; standard deviation = 1), and β was the standardized effect size.
